# Antibiotic Prescription Practices in Ophthalmology Outpatient Clinics in Khartoum, Sudan: An Observational Cross-Sectional Study

**DOI:** 10.7759/cureus.108474

**Published:** 2026-05-08

**Authors:** Imtinan Yousif, Alrumaisa Alhassan, Mohammed Hamed Ibrahium Badi, Yasir Abdalla

**Affiliations:** 1 Ophthalmology, University of Khartoum, Khartoum, SDN; 2 Pediatrics, University of Khartoum, Khartoum, SDN; 3 Education Development Centre, University of Khartoum, Khartoum, SDN; 4 Ophthalmology, Alwalidain Charity Hospital, Khartoum, SDN

**Keywords:** antibiotic prescription, antibiotics, antimicrobial resistance, case scenarios, ophthalmology clinics

## Abstract

Background: Antimicrobial resistance is a growing global health concern, posing serious threats to the population's health and well-being. However, the situation in Sudan is unknown. This study explores antibiotic prescriptions in ophthalmology outpatient clinics in Khartoum.

Methods: This descriptive cross-sectional study was conducted from January to April 2023 across major ophthalmology teaching hospitals in Khartoum. Data were collected using a validated, pretested, anonymous online questionnaire capturing demographic information and responses to five guideline-based clinical vignettes. Analysis was performed via SPSS Version 27 (IBM Corp., Armonk, NY, USA).

Results: Of the 215 participants, 64.7% (n=139) were prescribed using brand names, and only 12.6% (n=27) of them had attended a course on prudent antibiotic prescription. Scientific articles or guidelines were the primary sources for practice for 62.8% (n=135) of the total participants; yet, residents mainly depended on asking a senior colleague (42%, n=55). Regarding case-related practice questions, most of the participants complied with the guidelines for grade two hyphema (63.3%, n=136), subconjunctival hemorrhage (80.9%, n=174), and preoperative preparation for cataract surgery (63.7%, n=137). For acute postoperative endophthalmitis, 68.8% (n=148) of the participants used intravitreal antibiotic injections. However, there was no specific preference for certain antibiotics in the case of corneal abrasion.

Conclusion: The practice of antibiotic prescription in this study varied among respondents and from the literature. It also highlighted the impact of the work environment on prescription practice, which might be attributable to the deficiency of a unified source of evidence and practice nationally. Therefore, the development of national ophthalmic antibiotic guidelines, structured training, and periodic audits would improve practice patterns.

## Introduction

Drug utilization research, as defined by the World Health Organization (WHO), is research that evaluates the prescribing, marketing, distribution, and use of medications within a particular community, with a focus on the consequent medical, social, and economic implications [1]. Antibiotics are commonly subject to irrational prescribing [2], possibly due to commercial pressures and challenges in diagnosing and differentiating between bacterial and viral infections [3]. This leads to inadequate care, unnecessary financial burden, and the emergence of antimicrobial-resistant bacteria [2].

Antimicrobial resistance (AMR) is the growing resistance of infectious microorganisms to formerly effective antimicrobial treatments [3]. Recent clinical trials have confirmed that the efficacy of widely utilized ophthalmic antibiotics is gradually declining [4]. In addition to AMR, irrational ophthalmic antibiotic use can result in disruption of the normal flora in the eye, consequently increasing the risk of recurrent infections, especially in the pediatric population. It also has the potential to cause ocular surface toxicity [4]. Ocular infection treatment failure can lead to vision loss and blindness [5].

Over the past two decades, AMR has garnered significant attention in the public health domain [3]. This global increase in bacterial resistance has compromised the effectiveness of antibiotics, and the inappropriate and excessive use of these medications has imposed a significant economic burden, particularly in low- and middle-income countries like Sudan [6]. Research has revealed that 50% of prescribed antibiotics in Africa are either unnecessary or administered against established guidelines. Additionally, several African countries require significant improvements in the provision and execution of antimicrobial therapy stewardship (APS) programs [7].

Therefore, all healthcare professionals need to practice rationally to avoid these problems, highlighting the importance of regularly evaluating drug use patterns to enhance treatment efficacy and prevent the emergence of resistance [1].

When searching the literature, there is a noticeable lack of information about Sudan's antimicrobial surveillance program or antibiotic prescribing practices. Therefore, this study aims to describe antibiotic prescribing patterns, evaluate adherence to clinical guidelines, and compare practices across professional levels in ophthalmic outpatient clinics in Khartoum.

## Materials and methods

Population and sample 

This descriptive cross-sectional study was conducted between January and April 2023 in the main ophthalmology teaching hospitals in Khartoum State, Sudan, namely, Makkah Eye Complex, Walidain Charity Eye Hospital, and Khartoum Teaching Eye Hospital. A total of 251 doctors were actively working across the three hospitals, including 149 specialists and 102 residents; 215 participants were included in the study, with a subsequent response rate of 85.7%.

Inclusion criteria

All ophthalmology residents and board-certified ophthalmologists (or those holding an equivalent qualification) who were actively working at the study sites during the study period were included.

Exclusion criteria

Participants on extended leave, residents who had not commenced formal ophthalmology training, and those who refused to participate were excluded.

Data and sources of data

An online, structured, pretested, self-administered, anonymous questionnaire was distributed to all eligible doctors using an internet-based application (WhatsApp), with periodic reminders to maximize the response rate (Appendix A). The questionnaire underwent content validation by three ophthalmologists and a clinical pharmacist to ensure relevance and clarity, and face validity was confirmed through a pilot test on a sample of 10 doctors who were not included in the final analysis, with necessary modifications made based on their feedback. Demographic details and practice-related information were included in the questionnaire, in addition to questioning the respondents’ reported management approach to five common ophthalmological cases. Each case was presented as a multiple-choice question with four possible responses (coded numerically from one to four). The ideal responses were predetermined based on internationally approved guidelines, selected due to the lack of local guidelines [8-11].

Each case was scored as follows: a score of one was assigned if the respondent selected the ideal (guideline-compliant) answer, and zero otherwise. A composite “Practice Score” was then calculated for each respondent by summing the scores across the five cases, yielding a possible range from zero (no guideline-compliant responses) to five (all responses compliant).

Clinical cases with their guidelines-compliant answers

Grade II hyphema following blunt trauma, with otherwise normal clinical and investigative findings: not prescribing antibiotics was the guideline-compliant answer [8,9].

Subconjunctival hemorrhage after blunt trauma, which accompanied normal examination and investigation findings: not prescribing antibiotics was considered best practice [9,10].

Treatment for acute postoperative endophthalmitis: intravitreal antibiotics were the ideal treatment [9,11].

The usual treatment for noninfective, non-center-involving corneal abrasions that occurred after blunt trauma: antibiotic ointment was the preferred treatment of choice [9].

The usual preoperative preparation for patients undergoing cataract surgery: again, non-antibiotic prescription was considered best practice [9].

Statistical analysis

Data were cleaned in Microsoft Excel (Microsoft Corporation, Redmond, WA, USA) and analyzed using IBM SPSS Statistics Version 27 (IBM Corp., Armonk, NY, USA). A composite practice score (range 0-5) was computed for each participant by summing guideline-concordant case-based question responses. Descriptive statistics are presented as frequencies and percentages for categorical variables and mean±standard deviation (with range) for continuous variables. Between-group comparisons employed Pearson’s chi-square test (or the likelihood-ratio test when appropriate) for categorical outcomes, independent-samples t-tests for two-group comparisons of the practice score, and one-way ANOVA for comparisons across more than two groups with Tukey’s post hoc tests when ANOVA was significant. Multivariable analysis used binary logistic regression to identify independent predictors of the binary outcome of interest, with regression coefficients (B), standard errors, Wald statistics, p-values, odds ratios (Exp(B)), and Nagelkerke R² reported. All tests were two-tailed, a significance level of α=0.05 was used, and analyses were performed on the available sample with listwise deletion (valid N as reported).

Ethical approval

Ethical approval for the study was obtained from the Health Sector Ethical Review Committee, University of Gezira, Sudan (Number: 316/2022). Successfully filling out the questionnaire was considered participant consent.

## Results

The response rate was 85.7% (n=215); 79.1% of the participants were female (n=170). Residents constituted 60.9% (n=131) of the sample. Table 1 provides additional information on the demographic characteristics of the study population.

**Table 1:** Demographic characteristics of the study population

Variable	Frequency	Percentage
Gender		
Male	45	20.9%
Female	170	79.1%
Designation		
Resident	131	60.9%
Specialist	84	39.1%
Work place		
Khartoum Teaching Eye Hospital	93	43.3%
Makkah Eye Complex	69	32.1%
Walidain Charity Eye Hospital	53	24.6%

Overall, textbooks were the major source of information regarding antibiotic prescription (33.5% of all study participants, n=72). 42.0% (n=55) of residents relied on senior colleagues as their primary source of information, compared to 11.9% (n=10) of specialists/consultants. Conversely, 73.8% (n=62) of specialists used scientific resources (i.e., guidelines and textbooks), while only 55.7% (n=73) of residents did (Table 2).

**Table 2:** Factors influencing antibiotic prescription among different levels of clinical experience

Variable	Resident (% within residents)	Specialist or consultant (% within specialist or consultant)	Total (% of total)
Primary source of information			
Guidelines	34 (26.0%)	29 (34.5%)	63 (29.3%)
Pharmaceutical	3 (2.3%)	11 (13.1%)	14 (6.5%)
Senior	55 (42.0%)	10 (11.9%)	65 (30.2%)
Textbooks	39 (29.8%)	33 (39.3%)	72 (33.5%)
Conference	0 (0.0%)	1 (1.2%)	1 (0.5%)
Course attendance			
Yes	9 (6.9%)	18 (21.4%)	27 (12.6%)
No	122 (93.1%)	66 (78.6%)	188 (87.4%)
Prescribing pattern			
Generic name	55 (42%)	21 (25%)	76 (35.3%)
Brand name	76 (58%)	63 (75%)	139 (64.7%)
Total	131	84	215 (100%)

As we considered antibiotic prescription patterns, only 12.6% (n=27) of the participants had attended a course on prudent antibiotic prescription. Prescribing the brand name was the most common prescription pattern among all practitioners, at 64.7% (n=139). Table 2 provides a detailed description.

Regarding the case-based questions, the first aimed to evaluate the routine management of grade II hyphema following blunt trauma, with otherwise normal clinical and investigative findings. The majority of respondents, 63.3% (n=136), reported not prescribing antibiotics, which represented the predominant approach in both groups. The second was a question about subconjunctival hemorrhage after blunt trauma, which accompanied normal examination and investigation findings. Again, 80.9% (n=174) of the total participants reported not prescribing antibiotics, which was uniform across both grades of ophthalmologists.

When asked about the usual treatment for acute postoperative endophthalmitis, 68.8% (n=148) of the participants reported routinely using intravitreal antibiotic injections.

There was no common theme for the usual treatment in cases of noninfective, non-center-involving corneal abrasion that occurred after blunt trauma, as 29.3% (n=63) of all respondents used fusidic acid eye ointment, and 34.9% (n=75) used moxifloxacin eye drops, with varying preferences across groups.

Regarding preoperative preparation for patients undergoing cataract surgery, 63.7% (n=137) of the respondents reported that they do not usually prescribe antibiotics. However, approximately half of the residents reported their usual prescription of different types of antibiotics. Table 3 presents the details of these findings.

**Table 3:** Antibiotic selection in specified ophthalmic conditions among different levels of clinical experience Percentages are percentages of the total sample (N=215). ED, eye drops; EO, eye ointment

Case/choice	Resident (n=131)	Specialist (n=84)	Total count (N=215)
Grade 2 hyphema after blunt trauma, with normal examination and investigations			
Moxifloxacin ED	26 (12.1%)	14 (6.5%)	40 (18.6%)
Ciprofloxacin ED	16 (7.4%)	6 (2.8%)	22 (10.2%)
Tobramycin ED	12 (5.6%)	5 (2.3%)	17 (7.9%)
No antibiotic ED is needed	77 (35.8%)	59 (27.4%)	136 (63.3%)
Subconjunctival hemorrhage after blunt trauma, with normal examination and investigations			
Ciprofloxacin ED	13 (6.0%)	8 (3.7%)	21 (9.8%)
Moxifloxacin ED	8 (3.7%)	3 (1.4%)	11 (5.1%)
Tobramycin ED	9 (4.2%)	0 (0%)	9 (4.2%)
No antibiotic ED is needed	101 (47%)	73 (34%)	174 (80.9%)
Acute postoperative endophthalmitis			
Intravitreal injection	83 (38.6%)	65 (30.2%)	148 (68.8%)
Frequent moxifloxacin ED	25 (11.6%)	16 (7.4%)	41 (19.1%)
Intravenous antibiotics	18 (8.3%)	2 (0.9%)	20 (9.3%)
Frequent ciprofloxacin ED	5 (2.3%)	1 (0.4%)	6 (2.8%)
Blunt trauma causing corneal abrasion, which is noninfective/non-center involving			
Fusidic acid EO	37 (17.2%)	26 (12.1%)	63 (29.3%)
Moxifloxacin ED	49 (22.8%)	26 (12.1%)	75 (34.9%)
Ciprofloxacin ED	27 (12.6%)	23 (10.7%)	50 (23.3%)
No antibiotic ED is needed	18 (8.4%)	9 (4.2%)	27 (12.6%)
Preoperative preparation for cataract surgery			
Ciprofloxacin ED	17 (7.9%)	13 (6%)	30 (14%)
Moxifloxacin ED	26 (12.1%)	12 (5.6%)	38 (17.7%)
Tobramycin ED	7 (3.3%)	3 (1.4%)	10 (4.7%)
No antibiotic ED is needed	81 (37.7%)	56 (26%)	137 (63.7%)

Further analysis using logistic regression indicated that the specialists/consultants group had 2.19 times higher odds of using brand names compared to residents (p=0.013). Gender, workplace, and course participation did not significantly influence writing style (p>0.05). The model explained only 4.9% of the variance (Nagelkerke R²), suggesting limited predictive power. Table 4 presents the details of these findings.

**Table 4:** Logistic regression predictors of brand-name prescribing (writing style) Binary logistic regression; dependent variable coded as one = brand name prescribing, zero = generic name prescribing. Sample gender counts: male (n=45); female (n=170). Reference category for gender = female. Predictor p-values reported are from Wald tests. Model fit: Nagelkerke R²=0.049. SE, standard error

Predictor	B (coefficient)	SE	Wald	p-value	Exp(B) (odds ratio)
Gender (male)	-0.331	0.373	0.784	0.376	0.718
Position (specialist)	0.784	0.317	6.123	0.013	2.191
Workplace	-0.081	0.170	0.225	0.635	0.923
Course	0.118	0.459	0.067	0.796	1.126
Constant	0.080	1.331	0.004	0.952	1.084

## Discussion

This study provides an initial description of the patterns of topical antibiotic prescription by ophthalmologists in outpatient clinics in the main ophthalmology teaching hospitals in Khartoum.

While specialists constituted the main study population, the response rate was higher among residents; this may be due to their interest in the research and ability to use internet-based data collection methods.

Females constituted 79.1% (n=170) of the respondents, which is higher than the global ratio of females in ophthalmology, which is only 30% [12].

When we inquired about attending a course on prudent antibiotic prescription, the attendance rate was low among both residents and specialists. This may explain the nonsignificant statistical association between course attendance and the calculated case-based practice scores.

A significant number of residents reported relying on advice from a senior colleague as the primary source of usual antibiotic prescription; this might indicate that residents depend more on institutional knowledge (e.g., seniors), whereas specialists prioritize scientific evidence (e.g., guidelines and textbooks).

Prescribing a brand name was the predominant practice in both study populations. It was also the predominant practice (65%) at a major teaching hospital in India [13]; on the other hand, a Saudi study found that doctors mostly prescribe generic names; however, the commercial effect of pharmaceutical companies might influence the choice of the prescribed drug [14].

Regarding the clinical case questions, 63.3% (n=136) of the participants would not prescribe antibiotics for grade 2 hyphema following blunt trauma. This is considered best practice as outlined by a local NHS trust guideline and the Wills Eye Manual [8,9], respectively. This is in line with a previous study conducted by Chaddah MR and Gupta SP, which concluded that the use of 4% pilocarpine, as opposed to chloramphenicol eye ointment or a combination of atropine and chloramphenicol, results in the fastest recovery in grade 1 and 2 hyphema [15]. In addition to a study by Miller SC et al., where participants concluded that the use of topical steroids for gross and micro hyphema and topical cycloplegic medications, with atropine being the most popular [16].

In the case of subconjunctival hemorrhage, 80.9% (n=174) of respondents reported that they would not prescribe antibiotics. This practice is recommended by Holly Cronau et al., who found that subconjunctival hemorrhage recovers spontaneously over a few weeks [17]. Likewise, this is consistent with the Wills Eye Manual and a local NHS trust guideline [9,10], which we considered best practice.

In the case of postoperative endophthalmitis, most of the study participants stated that they would prescribe intravitreal injection (68.8%, n=148), as outlined by the Wills Eye Manual as well as a local NHS trust guideline [9,11]. However, the rest of the participants would prescribe less effective treatment modalities [18], which may endanger patients’ vision. Topical medical management combined with intravitreal vancomycin+ceftazidime was studied by Verma L et al., who concluded that aggressive therapy is advised to include immediate pars plana vitrectomy as well as intravitreal vancomycin and ceftazidime administration. Antibiotics that are effective against likely organisms should also be used topically and systemically [19].

Constance W et al. stated that the best course of action for treating acute endophthalmitis is intravitreal antibiotic administration, which is the cornerstone of treatment to stop the infection and protect vision. In situations where there is low visual acuity, such as light perception or worse, immediate vitrectomy is beneficial [20]. In their update, Althiabi S et al. argued that the combination of intravitreal injections of unpreserved dexamethasone and broad-spectrum antibiotics is key to managing massive bacterial postoperative endophthalmitis. Intravitreal injections of vancomycin-ceftazidime are used as the first line of treatment, while vancomycin-amikacin is the second [21].

In the case of noninfectious, noncentral corneal abrasion, there was no common theme in treatment options chosen by our participants; however, antibiotic ointment was the preferred treatment of choice according to the Wills Eye Manual [9] because it serves as a barrier and lubricant between the eyelid and abrasion. In a review article by Dang DH et al., the use of topical antibiotics was found to be unnecessary unless a large abrasion or a foreign body was involved, or a corneal epithelial defect was present to prevent secondary infections [22]. A Cochrane Library review by Algarni AM et al. concluded that there is insufficient evidence to support using antibiotic prophylaxis to prevent ocular infection or accelerate healing [23].

Regarding preoperative preparation for patients undergoing cataract surgery, 63.7% (n=137) of the respondents did not prescribe any antibiotics. The Wills Eye Manual [9] confirms that the use of antibiotics before cataract surgery might decrease the bacterial load; however, it will not decrease the infection risk, yet it increases the possibility of consequent development of AMR. On the other hand, a survey that included 26 eye institutions in Asia by Garg P et al. found that two-thirds of the respondents reported using preoperative antibiotic prophylaxis and specified using topical levofloxacin for patients with low risk of infection and moxifloxacin for high-risk patients [24]. In an RCT in Brazil by Tiago Eugnio Faria and Arantes et al., they found that applying antibiotic prophylaxis has no statistical significance compared to povidone-iodine [25].

The scope of our study focused more on antibiotic prescription rather than complete management practice, since it is the most important aspect of AMR.

Limitations

The study's reliance on self-reported data introduces potential reporting bias, as participants may report what they perceive as ideal rather than their actual practice at hospitals; however, due to documentation deficiency, it was not feasible to directly study the presentations. The relatively small sample size reduces the generalizability of the study. Additionally, being a descriptive cross-sectional study limits the ability to establish causal relationships between prescribers’ characteristics and their prescribing patterns; however, this study provides valuable baseline data and highlights gaps in preexisting patterns.

Recommendations

There is a pressing need for a unified guideline to regulate antibiotic prescription practice in ophthalmology in Sudan, and we hope that this study draws attention to this issue and eventually leads to published evidence-based guidelines to regulate practitioners' antibiotic prescription patterns, in addition to encouraging regular audit and feedback mechanisms and targeted training programs to improve prescribing practices.

## Conclusions

This baseline study assessed antibiotic prescription practices among ophthalmologists in outpatient clinics across major teaching hospitals in Khartoum to identify gaps in rational antibiotic use. The results highlighted the effect of the work environment on prescription practices, which might be attributable to the deficiency of a unified source of evidence and practice nationally.

Multiple gaps were identified, including considerable variation in prescription patterns, limited adherence to evidence-based guidelines, and reliance of most practitioners on non-standardized sources of prescription practices, such as asking senior colleagues. Additionally, our findings revealed that only a few participants had received formal evidence-based training on the rational use of antibiotics; this may partly explain the observed variations in prescribing practices and underscores the need for wider implementation of educational initiatives. Additionally, the development of national ophthalmic antibiotic guidelines and periodic audits could improve practice patterns.

## Appendices

Appendix A

**Table 5:** Questionnaire

Question number	Question	Options
1	Gender	A. Male B. Female
2	Your position	A. Registrar B. Specialist (1-7 years) C. Consultant (>7 years ophthalmologist)
3	Workplace before the war	A. Makka Eye Hospital B. Walidain Charity Eye Hospital C. Khartoum Eye Hospital
4	Source of information on antibiotics you use (MAINLY)	A. Scientific articles/guidelines B. Pharmaceutical medical representative C. From a senior D. Medical textbook E. Other (specify)
5	In your daily practice, do you prescribe using the generic name or the brand name	A. Generic name (e.g., moxifloxacin) B. Brand name (e.g., Vigamox, Fortymox, Oxcin)
6	Have you attended a course in the appropriate antibiotics practice?	A. Yes B. No
Scenarios (please choose the answer that reflects your usual practice)		
1	In a case of grade 2 hyphema after blunt trauma; rest of the examinations were NORMAL. Your management includes:	A. Moxifloxacin ED (Fortymox, Vigamox, Oxcin) B. Ciprofloxacin ED (e.g., Ciloxan, Opticin) C. Tobramycin ED (e.g., Tobrex, Tobramax, Bracin) D. No antibiotic ED is needed
2	In a case of blunt trauma causing subconjunctival hemorrhage with otherwise NORMAL examinations and investigations, your management includes:	A. Ciprofloxacin ED (e.g., Ciloxan, Opticin) B. Moxifloxacin ED (Fortymox, Vigamox, Oxcin) C. Tobramycin ED (e.g., Tobrex, Tobramax, Bracin) D. No antibiotic ED is needed
3	In a case of acute simple bacterial conjunctivitis (non-gonococcal/non-chlamydial), your management includes:	A. Ciprofloxacin ED (e.g., Ciloxan, Opticin) B. Gatifloxacin ED (e.g., Tymer, Gatiflox, Gatistar) C. Moxifloxacin ED (Fortymox, Vigamox, Oxcin) D. Tobramycin ED (e.g., Tobrex, Tobramax, Bracin)
4	In a case of acute post-operative endophthalmitis, your IMMEDIATE management includes:	A. Intravenous antibiotics injections B. Frequent moxifloxacin ED C. Intravenous antibiotics injections D. Frequent ciprofloxacin ED
5	In a case of blunt trauma causing simple non-infective/non-central corneal abrasion (no history of contact lens wear), your management includes:	A. Fucidic acid eye ointment (e.g., Optifucin EO) B. Moxifloxacin eye drop (e.g., Fortymox, Vigamox, Oxcin) C. Ciprofloxacin eye drop (e.g., Ciloxan ED, Opticin ED) D. No antibiotic ED is needed
6	Preoperative preparation for a patient undergoing cataract extraction surgery:	A. Ciprofloxacin eye drop (e.g., Ciloxan ED, Opticin ED) B. Moxifloxacin eye drop (e.g., Fortymox, Vigamox, Oxcin) C. Tobramycin ED (e.g., Tobrex, Tobramax, Bracin) D. No antibiotic ED is needed
